# Clinical characteristics and preliminary retrospective evidence of adjunctive efficacy of intense pulsed light therapy for Demodex-associated blepharokeratoconjunctivitis: a single-center study

**DOI:** 10.1080/07853890.2026.2639675

**Published:** 2026-03-08

**Authors:** Shuo Yang, Ning Wang, Qingli Meng, Lin Lin, Lijuan Wang, Xiuming Jin

**Affiliations:** ^a^ Eye Center of Second Affiliated Hospital, School of Medicine, Zhejiang University, Hangzhou, China; ^b^ Zhejiang Provincial Key Laboratory of Ophthalmology, Zhejiang Provincial Clinical Research Center for Eye Diseases, Zhejiang Provincial Engineering Institute on Eye Diseases, Hangzhou, China; ^c^ Department of Ophthalmology, Affiliated Hospital of Jining Medical University, Jining, China

**Keywords:** *Demodex* blepharokeratoconjunctivitis, intense pulsed light therapy, meibomian gland dysfunction, corneal neovascularization, adjunctive therapy

## Abstract

**Objective:**

To characterize clinical features of Demodex blepharokeratoconjunctivitis (BKC) and evaluate the efficacy of intense pulsed light (IPL) adjunctive therapy.

**Methods:**

Retrospective cohort study of 45 microbiologically confirmed Demodex-BKC patients. Outcomes were compared between conventional treatment (*n* = 35) and IPL-adjunctive groups (*n* = 10).

**Results:**

Corneal lesions predominantly involved the inferior cornea, with characteristic lid margin signs including cylindrical dandruff and recurrent chalazia. Significant diagnostic delays and frequent misdiagnosis were observed. Higher Demodex density strongly correlated with increased corneal lesion severity, central corneal involvement, and worse meibomian gland dysfunction. IPL adjunctive therapy demonstrated significantly superior outcomes *versus* conventional treatment: greater neovascularization regression, improved tear film stability, better visual acuity gain, and reduced 6-month recurrence. Treatment was well-tolerated without adverse events.

**Conclusion:**

In this retrospective study, IPL adjunctive therapy was associated with improved clinical outcomes in Demodex-BKC, including reductions in mite burden, inflammation, and recurrence risk. These findings suggest it may represent a promising adjunctive therapeutic strategy.

## 1. Introduction

Blepharitis-associated keratoconjunctivitis (BKC) refers to a group of keratoconjunctival disorders associated with blepharitis, characterized by recurrent conjunctivitis and corneal lesions, including corneal epithelial erosion, punctate keratitis, marginal keratitis, and corneal ulceration. Clinically, BKC is often misdiagnosed or managed with delay, posing a significant threat to patients’ visual health [1,2].

Blepharitis, as a primary etiological factor of BKC, can itself be induced by multiple factors. These include non-infectious factors such as chronic refractive errors, use of electronic devices, high-fat diet, high-sugar diet, dry environment, air pollution, contact lens wear, sex hormone deficiency, aging, hypercholesterolemia, antidepressants, antihistamines, antihypertensive medications, and postmenopausal hormone therapy [3–12]. Notably, these non-infectious factors not only serve as major causes of meibomian gland dysfunction (MGD) but also predispose to blepharitis. Importantly, disruption of the eyelid margin ocular surface barrier caused by non-infectious factors and MGD creates a ‘fertile soil’ for the proliferation of infectious agents. Nowadays, particularly in patients with refractory blepharitis or MGD, the presence of infectious factors should be considered. Among these, Demodex mite infection has emerged as a critical contributor to infectious blepharitis [13,14].

Demodex mites are the most common microscopic ectoparasites found in human skin. Their prevalence increases with age, with 84% of individuals aged 60 years and 100% of those over 70 years affected [13]. The unique anatomical structure of the eye, combined with residual cosmetics on eyelashes and challenges in eyelid hygiene, facilitates Demodex proliferation. Studies suggest that the incidence of Demodex-associated blepharitis is 29–90%, accounting for 60% of all blepharitis cases [15–19]. Although the positive rate of Demodex infection in the general population is considerable, not all infections lead to blepharitis. However, when heavy accumulation of secretions or excreta occurs at the base of eyelashes and eyelid margins, accompanied by eyelid margin inflammation, Demodex-induced blepharitis should be suspected [15,20,21]. Recurrent or persistent blepharitis further progresses to keratoconjunctivitis, impairing visual health.

Demodex mites cause direct damage to the eyelid margin and induce immune responses. Recurrent inflammatory and infectious stimuli contribute to inflammatory damage in the cornea and conjunctiva. There is growing recognition of the role of Demodex infection in refractory keratitis, and targeted demodicidal therapy has been reported to be associated with improved outcomes and prognosis in refractory BKC [23]. Previous studies on Demodex-associated BKC have lacked focused investigations into clinical characteristics, and treatment approaches remain relatively limited. Current protocols primarily follow conventional BKC management, supplemented with topical tea tree oil or pine turpentine oil for demodicidal therapy, yet these methods show relatively limited long-term efficacy.

Intense pulsed light (IPL) has emerged as a novel therapeutic modality for MGD and blepharitis, offering unique advantages. Its physical photothermal effect directly targets meibomian glands and eyelid margins, occluding pathologic neovessels and alleviating local inflammation with rapid and sustained efficacy [24]. IPL is often integrated into comprehensive treatment regimens for blepharitis and MGD, such as combined with topical demodicidal agents (tea tree oil), artificial tears, or immunosuppressants, thereby enhancing overall therapeutic outcomes. Additionally, IPL improves the ocular surface microenvironment, reducing reliance on corticosteroids [24]. Prior studies, including those by Fang Ruan et al. have demonstrated that IPL significantly alleviates symptoms of Demodex-associated blepharitis and improves clinical outcomes in BKC patients [25]. Other investigations have shown that IPL suppresses ocular surface inflammatory cytokines, softens meibum, enhances glandular secretion, and directly disrupts the breeding environment of Demodex mites, exerting inhibitory effects on their survival [26,27].

Although some studies have explored IPL applications in ocular diseases, there remains a paucity of research comparing conventional treatment with IPL-combined therapy in terms of symptom improvement, corneal lesion repair, and long-term prognosis (recurrence rate) specifically for Demodex-infected blepharitis-associated keratitis. This study aims to conduct a retrospective analysis of clinical data from such patients to investigate the clinical characteristics of Demodex-infected blepharitis-associated keratitis, compare the efficacy of conventional *versus* IPL-combined therapy, evaluate the potential of IPL combination therapy, and provide evidence for optimizing clinical treatment protocols.

## 2. Materials and methods

### 2.1. Study design and case enrollment

This retrospective cohort study enrolled a consecutive series of 45 patients clinically diagnosed with Demodex‑infected blepharitis complicated by keratitis, identified from the outpatient electronic medical record database of the Cornea and Ocular Surface Disease Specialist Center, Second Affiliated Hospital of Zhejiang University School of Medicine. Data collection spanned from January 2022 to January 2024, and the retrospective analysis was conducted between September and October 2025. Although the overall design is a retrospective cohort study, the analysis of associations between baseline characteristics (Demodex density and corneal lesion severity) is cross‑sectional in nature. Based on treatment modalities, patients were divided into two groups: the conventional treatment group and the IPL‑combined therapy group. The study was conducted in accordance with the Declaration of Helsinki and approved by the Institutional Review Board of the Second Affiliated Hospital of Zhejiang University. As a retrospective analysis of de-identified clinical data involving no additional interventions, and given the geographically dispersed cohort for whom recall for written consent was not feasible, the IRB approved the use of verbal informed consent. Trained research coordinators contacted eligible patients (or their guardians) to explain the study purpose, the use of anonymized data, and the right to withdraw; verbal agreement was documented in a standardized study log. For patients whose clinical images are included, separate written informed consent for publication was obtained.

### 2.2. Patient allocation to treatment groups

Assignment to the IPL-combined therapy group or the conventional treatment group was not randomized but based on integrated clinical decision-making in real-world practice. The decision primarily considered the following factors: (1) Disease refractoriness: Patients with frequent recurrences or inadequate response to prior conventional therapies were more likely to be counseled about IPL as an additional therapeutic option; (2) Informed patient preference and willingness: After detailed discussion regarding the potential benefits, procedure details, and out-of-pocket costs of IPL, treatment choice was ultimately made by the patient; (3) Device availability during the patient’s treatment period.

### 2.3. Microbiological (Demodex) examination

All enrolled patients tested positive for microbiological detection of Demodex. Among them, 36 cases (80%) were confirmed *via* light microscopy, while 9 cases (20%) were diagnosed using confocal microscopy. The primary reasons for patient refusal of eyelash sampling included subjective apprehensions such as fear of pain/discomfort and concerns about potential impacts on lash growth and aesthetics.

#### 2.3.1. Light microscopy examination

For each eyelid, 3 eyelashes (preferably those with lipid-like sleeve-shaped secretions at the root, or misdirected/curled eyelashes) were collected, totaling 12 eyelashes from both upper and lower eyelids of both eyes. The plucked eyelashes were placed parallel on a glass slide, covered with cedarwood oil, and examined under a light microscope to count and characterize Demodex mites [25].

#### 2.3.2. In vivo *confocal microscopy (IVCM) examination*

This method allows rapid detection of multiple hair follicles *in vivo*, along with observation of adjacent eyelid margin and meibomian gland structures, as well as Demodex infection within meibomian glands. It remains applicable for Demodex detection even in patients with partial eyelash loss.

### 2.4. Diagnostic criteria

#### 2.4.1. Demodex-associated blepharitis

The diagnosis of *Demodex* blepharitis was established based on the following criteria [28]: (1) a chronic or subacute clinical course with bilateral ocular symptoms, such as redness, pruritus, or foreign body sensation, and/or a history of recurrent or refractory chalazia; (2) characteristic eyelid abnormalities, including the presence of lipid-like cylindrical dandruff (collarettes) around the base of the eyelashes—a pathognomonic sign—often accompanied by eyelid margin hyperemia and thickening; and (3) a positive *Demodex* test, defined as the detection of ≥3 mites per 3 eyelashes from any single eyelid in adult patients. All life stages of *Demodex* were counted toward the total. Cases with counts below this threshold were considered suspected positives and required correlation with clinical features, with further microbiological testing recommended to exclude other pathogens, such as bacteria or fungi.

A definitive diagnosis requires fulfillment of all three criteria. In cases where the first two clinical criteria are met but mite counts remain below the threshold, repeat sampling or *in vivo* confocal microscopy is recommended for confirmation. If counts persist below the diagnostic cutoff, a classification of ‘suspected *Demodex* blepharitis’ may be applied. Conversely, the detection of *Demodex* mites in the absence of clinical signs or symptoms does not support a diagnosis of *Demodex* blepharitis.

#### 2.4.2. Blepharitis-associated BKC

Blepharitis-associated corneal lesions are characterized by inflammatory involvement of the cornea, presenting as punctate epithelial erosions, stromal infiltrates, or ulcerations, frequently accompanied by superficial neovascularization. In severe cases, corneal ulcers may progress to perforation. The diagnosis of blepharitis-related keratopathy requires the following [29]: (1) presence of concurrent blepharitis; (2) objective evidence of corneal involvement, such as epithelial erosion, punctate keratitis, marginal keratitis, or ulceration; and (3) exclusion of alternative etiologies, including trauma, drug-induced toxicity, or viral/bacterial infection. Characteristically, these corneal infiltrates or ulcers are associated with a corneal pannus that extends from the limbus toward the lesion, with the inflammatory focus typically situated at the leading edge of the pannus. In early or initial presentations, lesions tend to be located in the corneal periphery; however, in cases of recurrence or chronicity, the disease may progress centripetally with accompanying pannus ingrowth toward the visual axis.

### 2.5. Clinical assessment for diagnosis and phenotyping

To fulfill the diagnostic criteria for Demodex-associated BKC (Section 2.4) and to phenotype the enrolled cohort, all patients underwent a standardized slit-lamp biomicroscopy evaluation. This evaluation systematically documented: (1) eyelid margin characteristics, including the presence of cylindrical dandruff (collarettes), hyperemia, and telangiectasia; (2) signs of meibomian gland dysfunction (MGD), assessed by secretion quality upon digital expression and gland morphology *via* infrared meibography; and (3) ocular surface and corneal lesion features, encompassing conjunctival hyperemia, corneal lesion type, extent based on quadrant involvement, and the presence of neovascularization. These assessments provided the objective clinical evidence required for diagnosis and established the baseline metrics for subsequent severity correlation analyses.

### 2.6. Corneal lesion extent scoring

Corneal lesion extent was scored on a 0‑5 scale according to the number of corneal quadrants affected: 0 (no lesion), 1 (<1 quadrant), 2 (¼–½ quadrant), 3 (½–¾ quadrant), 4 (>¾ quadrant), and 5 (corneal center involvement).

Other routine ophthalmic examinations included intraocular pressure (IOP, measured *via* air-puff Tono-Pen tonometer or an ICARE rebound tonometer), best-corrected visual acuity (BCVA), and anterior segment optical coherence tomography angiography (OCTA) in selected patients.

### 2.7. Management

#### 2.7.1. Note on treatment paradigms

In this study, both groups received identical standardized pharmacotherapy and basic eyelid hygiene (detailed in Section 2.7.2 and Table 1). The key difference lay in the choice of physical intervention: the conventional treatment group received traditional meibomian gland heating and massage after acute inflammation was controlled, while the IPL-combined therapy group received intense pulsed light (IPL) treatment. Within the treatment paradigm, IPL was conceptualized as a novel, integrated physical modality designed to achieve goals similar to traditional massage (glandular expression and anti-inflammatory effects) while potentially offering additional benefits such as direct acaricidal activity and vascular modulation. Therefore, the comparison in this study essentially evaluates the clinical outcomes of two distinct physical treatment strategies—‘traditional physical therapy (massage)’ *versus* ‘emerging physical therapy (IPL)’—on top of an identical pharmacological foundation.

**Table 1.** Summary and comparison of treatment protocols.

Treatment component	Conventional treatment group	IPL-combined therapy group	Notes on consistency/difference
Primary intervention	No IPL therapy	Received IPL therapy (M22 device, 15–24 J/cm²)	Core interventional difference.
Core medical therapy (identical)			All topical medications and basic eyelid hygiene were identical between groups.
• Topical corticosteroids	0.1% Fluorometholone/Loteprednol eye drops (QID, weekly taper)	Same as left	None
• Artificial tears	Non-anti-fogging artificial tears (QID)	Same as left	None
• Antibiotic-steroid ointment	Tobramycin/Dexamethasone ointment (applied to lid margin, BID-TID, intermittent use after 1 week)	Same as left	None
• Nighttime antibiotic	Ofloxacin eye ointment at bedtime (QHS)	Same as left	None
• Eyelid hygiene	Tea tree oil-based demodicidal wipes (BID first month)	Same as left	None
• Eyelid margin cleansing	Adjusted based on clinical signs of debris at follow-up, with the same criteria applied to both groups.	Same as left	Performed as needed in both groups according to the same clinical indications.
Potential variance (clarified)	Meibomian gland heating and massage (initiated after acute inflammation control)	Not systematically performed	This difference is attributed to the inherent photothermal effect of IPL, which provides targeted glandular therapy, as discussed.

IPL: intense pulsed light; BID: twice daily; TID: three times daily; QID: four times daily; QHS: at bedtime.

*Notes:* The core medical therapy (topical corticosteroids, artificial tears, antibiotic-steroid ointment, nighttime antibiotic, and tea tree oil lid hygiene) was identical in both treatment groups. The primary interventional difference was the application of IPL in the combined therapy group. Potential variances in eyelid margin cleansing and meibomian gland therapy are acknowledged and discussed in the context of their clinical rationale.

#### 2.7.2. Treatment medications

For patients with positive eyelid Demodex microbial detection and confirmed blepharitis-associated keratitis, 0.1% fluorometholone or loteprednol eye drops were administered four times daily (QID), with gradual dose reduction weekly until discontinuation after 1 month. Concurrently, non-anti-fogging artificial tears were given QID, and ofloxacin eye ointment was applied topically at bedtime. For patients with severe keratitis, tacrolimus eye drops were prescribed twice daily (BID). Tobramycin and dexamethasone eye ointment (Alcon, USA) was applied to the eyelid margin 2–3 times daily, with intermittent use every other day after 1 week. After 1 month of continuous local treatment, 0.05% cyclosporine eye drops and artificial tears were used for maintenance therapy. Additionally, tea tree oil-based demodicidal wipes were recommended for eyelid margin cleaning, with a maintenance regimen of twice daily (BID) in the first month and once daily (QD) in the second month (Table 1).

#### 2.7.3. Physical therapy

##### 2.7.3.1. Eyelid margin cleansing

When excessive sleeve-shaped debris or secretions accumulated at the base of eyelashes (based on clinical signs), eyelid margin cleansing was performed. Eyelid margin cleansing was performed using a sterile sponge brush (Mu-Shua brand; Huizhou Huayang Medical Device Co., Ltd., China) coupled with an ophthalmic handpiece (model DC-01; Wuhan Epoch Sunshine Technology Development Co., Ltd., China). The device consists of an electric handle and a cleaning brush head. High-speed brushing with the device removes blockages such as gland orifice crusting, lipid plugs, scales, skin debris, and bacterial metabolites, thereby opening the meibomian gland orifices.

##### 2.7.3.2. Meibomian gland therapy

During the acute phase of keratitis, meibomian gland therapy was not prioritized. For non-IPL-treated patients, after 1–2 weeks of drug therapy, when acute corneal inflammation was controlled, and corneal epithelial defects had significantly healed, meibomian gland heating and massage were performed.

##### 2.7.3.3. IPL therapy

**Figure 1.**
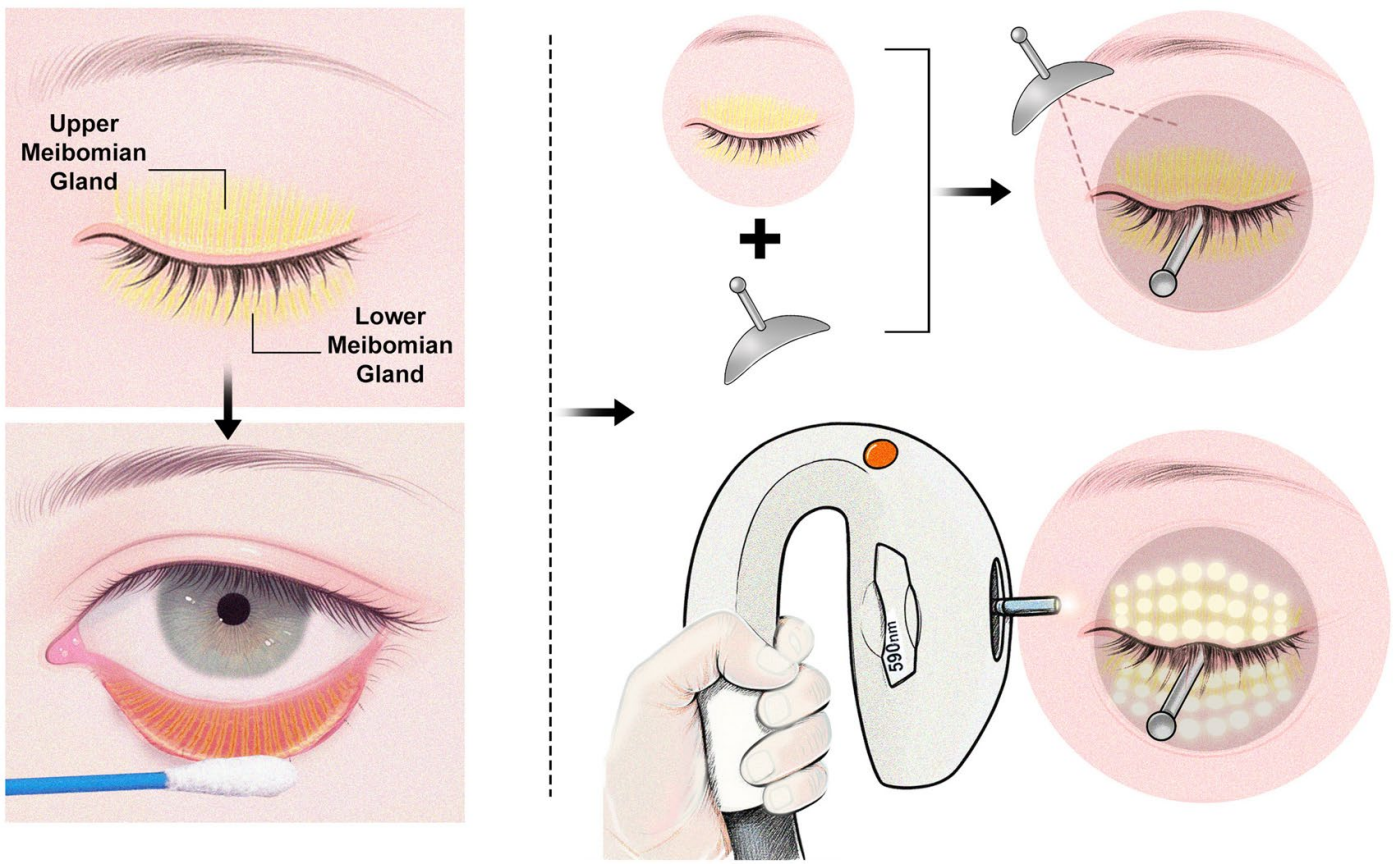
Schematic diagram demonstrating the application of the intense pulsed light (IPL) small handpiece in the periorbital area.

Among the 10 patients receiving IPL treatment, the M22 (OPT, M22™, Lumenis, USA) device was used with a small light guide contact head. For the small circular handpiece, the initial energy density was set at 15 J/cm^2^, with increments at each subsequent session, typically not exceeding 24 J/cm^2^.The procedure steps are briefly described as follows: First, the eyelid margin was cleaned with normal saline using a cotton swab. After instilling oxybuprocaine eye drops, an eye shield was placed, and light guide gel was applied to the eyelids. Sequential treatment was performed on the upper and lower eyelids using the small light guide head (see schematic diagram in Figure 1).

### 2.8. Statistical analysis

All statistical analyses were performed using SPSS software (Version 26.0, IBM Corp.). Continuous variables were first assessed for normality using the Shapiro‑Wilk test and for homogeneity of variances using Levene’s test. Data that met both normality and homoscedasticity assumptions are presented as mean ± standard deviation and were compared between two groups using the independent‑samples *t*‑test or among multiple groups using one‑way analysis of variance (ANOVA). Data that violated these assumptions are presented as median (interquartile range, IQR) and were compared using the Mann‑Whitney *U* test (two groups) or the Kruskal‑Wallis *H* test (multiple groups). Categorical variables are presented as frequency (percentage). For comparisons between two groups (2 × 2 contingency tables), Fisher’s exact test was applied when any expected cell count was < 5; otherwise, the chi‑square test was used. For comparisons involving more than two groups (R × C tables, where R > 2 or C > 2), the Fisher‑Freeman‑Halton exact test (multi‑group exact test) was employed if more than 20 % of expected cell counts were < 5 or any expected count was < 1; otherwise, the chi‑square test was used. To explore the association between Demodex density and central corneal involvement, a univariable logistic regression model was employed to calculate the crude odds ratio (OR) and its 95 % confidence interval (CI). Given the limited total sample size (*n* = 45), multivariable adjustment was not performed to avoid model overfitting. All tests were two‑tailed, and a *p*‑value <0.05 was considered statistically significant.

## 3. Results

### 3.1. Demographic characteristics and medical history

A total of 45 microbiologically confirmed Demodex-associated BKC patients were enrolled in this study, including 34 females (75.6%) and 11 males (24.4%). The overall mean age was 35.2 ± 18.6 years. Patients were divided into two treatment groups: the conventional treatment group (*n* = 35) and the IPL‑combined therapy group (*n* = 10). There was no significant difference in mean age between the conventional group (35.7 ± 19.5 years) and the IPL‑combined group (34.0 ± 13.9 years, *p* = 0.865). The most common presenting symptoms were persistent ocular redness and foreign body sensation (71.1%, 32/45), and recurrent chalazia were present in more than half of the patients (57.8%, 26/45). The median diagnostic delay from symptom onset to definitive diagnosis was 1.5 years (interquartile range: 0.3–4.8 years), with no significant difference between the two groups (*p* = 0.18). Initial misdiagnosis was observed in 37.8% (17/45) of patients: 22.2% (10/45) were misdiagnosed as viral keratitis and 15.6% (7/45) as simple blepharitis. A total of 31.1% (14/45) of patients received unnecessary antiviral treatment before correct diagnosis. Bilateral ocular involvement was noted in 60.0% (27/45) of cases, with a numerically higher proportion in the IPL‑combined group (70.0%) compared to the conventional group (57.1%), though this difference was not statistically significant (*p* = 0.49). Baseline Demodex density (mean mites per 3 eyelashes) was 3.8 ± 3.1 overall, with values of 3.6 ± 2.9 in the conventional group and 4.5 ± 3.6 in the IPL‑combined group (*p* = 0.43). At baseline, corneal neovascularization was present in 55.6% (25/45) of patients and central corneal involvement in 31.1% (14/45). The IPL‑combined group showed a higher baseline prevalence of both corneal neovascularization (80.0 *vs.* 48.6%, *p* = 0.08) and central corneal involvement (50.0 *vs.* 25.7%, *p* = 0.15) compared to the conventional group. Baseline corneal lesion extent scores were similar between groups (overall 2.1 ± 0.9; conventional 2.0 ± 0.9 *vs.* IPL‑combined 2.3 ± 0.8, *p* = 0.29). Detailed demographic and clinical baseline characteristics are summarized in Table 2.

**Table 2.** Demographic and clinical characteristics of enrolled patients.

Variable	Total patients (*n* = 45)	Conventional treatment group (*n* = 35)	IPL-combined therapy group (*n* = 10)	*p*-Value
Age (years)	38.7 ± 20.2	35.7 ± 19.5	34.0 ± 13.9	0.865
Sex, *n* (%)				
Female	34 (75.6)	26 (74.3)	8 (80.0)	Fisher-Freeman-Halton exact test, *p* = 1.000
Male	11 (24.4)	9 (25.7)	2 (20.0)	
Main symptoms, *n* (%)				
Ocular redness/foreign body sensation	32 (71.1)	–	–	–
Major comorbidities, *n* (%)				
Recurrent chalazia	26 (57.8)	–	–	–
Dry eye syndrome	28 (62.2)	–	–	–
Bilateral involvement, *n* (%)	27 (60.0)	20 (57.1)	7 (70.0)	Fisher-Freeman-Halton exact test, *p* = 0.726
Demodex density (mites/3 eyelashes), mean ± *SD*	3.8 ± 3.1	3.6 ± 2.9	4.5 ± 3.6	0.430
Corneal neovascularization (baseline), *n* (%)	25 (55.6)	17 (48.6)	8 (80.0)	Fisher-Freeman-Halton exact test, *p* = 0.161
Central corneal involvement (baseline), *n* (%)	14 (31.1)	9 (25.7)	5 (50.0)	Fisher-Freeman-Halton exact test, *p* = 0.245
Corneal lesion extent score (baseline), mean ± *SD*	2.1 ± 0.9	2.0 ± 0.9	2.3 ± 0.8	0.290
Previous misdiagnoses, *n* (%)				
Viral keratitis	10 (22.2)	–	–	–
Simple blepharitis	7 (15.6)	–	–	–
Diagnostic delay*	1.5 (0.3–4.8)	1.3 (0.3–4.0)	1.8 (0.4–5.2)	0.180
Antiviral drug use, *n* (%)	14 (31.1)	–	–	–

* Diagnostic delay refers to the time interval from the first onset of symptoms to the definitive diagnosis of *Demodex*-associated BKC.

### 3.2. Slit-lamp biomicroscopic findings: corneal lesion types, characteristics, and extent

**Figure 2.**
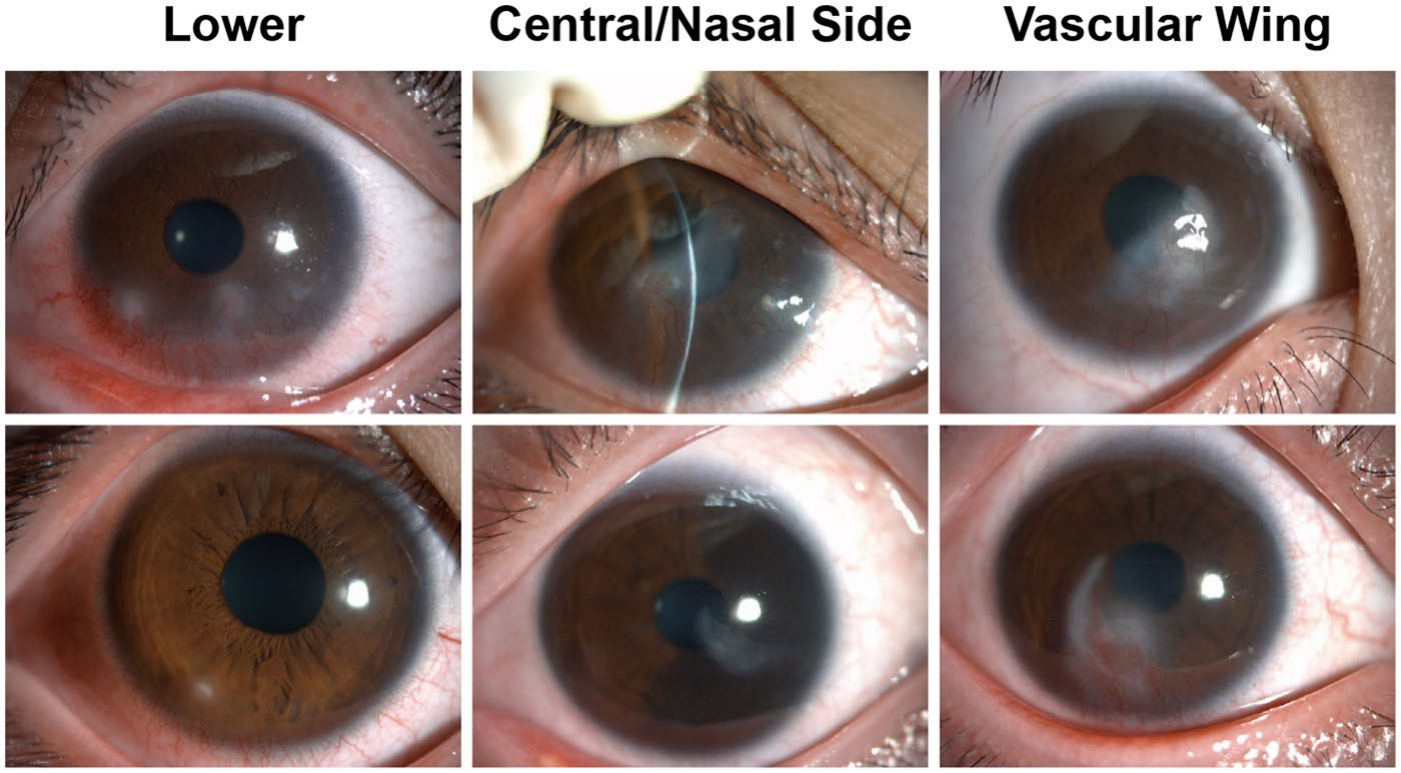
Anterior segment photographs of corneal damage in several representative cases of Demodex-related blepharokeratoconjunctivitis (BKC), showing lesions in the inferior, peripheral, central, and nasal cornea, as well as pannus formation.

Slit-lamp biomicroscopy revealed specific corneal manifestations of Demodex-associated BKC (Table 3). Regarding lesion distribution, 57.8% (26/45) of cases primarily involved the inferior cornea, which was significantly more common than other regions (*χ*^2^ = 12.7, *p* < 0.001); 26.7% (12/45) were located in the central/nasal cornea. Figure 2 shows the characteristic corneal damage associated with Demodex-induced blepharokeratoconjunctivitis (BKC).

**Table 3.** Slit-lamp biomicroscopic features of corneal lesions.

Clinical characteristic	*n* (%)
Predominant location	
Inferior cornea	26 (57.8)
Central/nasal cornea	12 (26.7)
Other locations	7 (15.6)
Lesion type	
Subepithelial/superficial stromal infiltration	28 (62.2)
Corneal nebula/macula	10 (22.2)
Ulcerative lesions	4 (8.9)
Associated neovascularization (Pannus)	25 (55.6)
└ Pannus invading central cornea	10 (40.0)

In terms of lesion types, subepithelial/shallow stromal infiltration was the most prevalent (62.2%, 28/45), typically presenting as grayish-white opacity in the peripheral or mid-peripheral cornea. Ulcerative lesions accounted for 8.9% (4/45), mostly occurring in the peripheral cornea with a clean base and no purulent exudation. Corneal nebula or macula developed in 22.2% (10/45) of cases.

**Figure 3.**
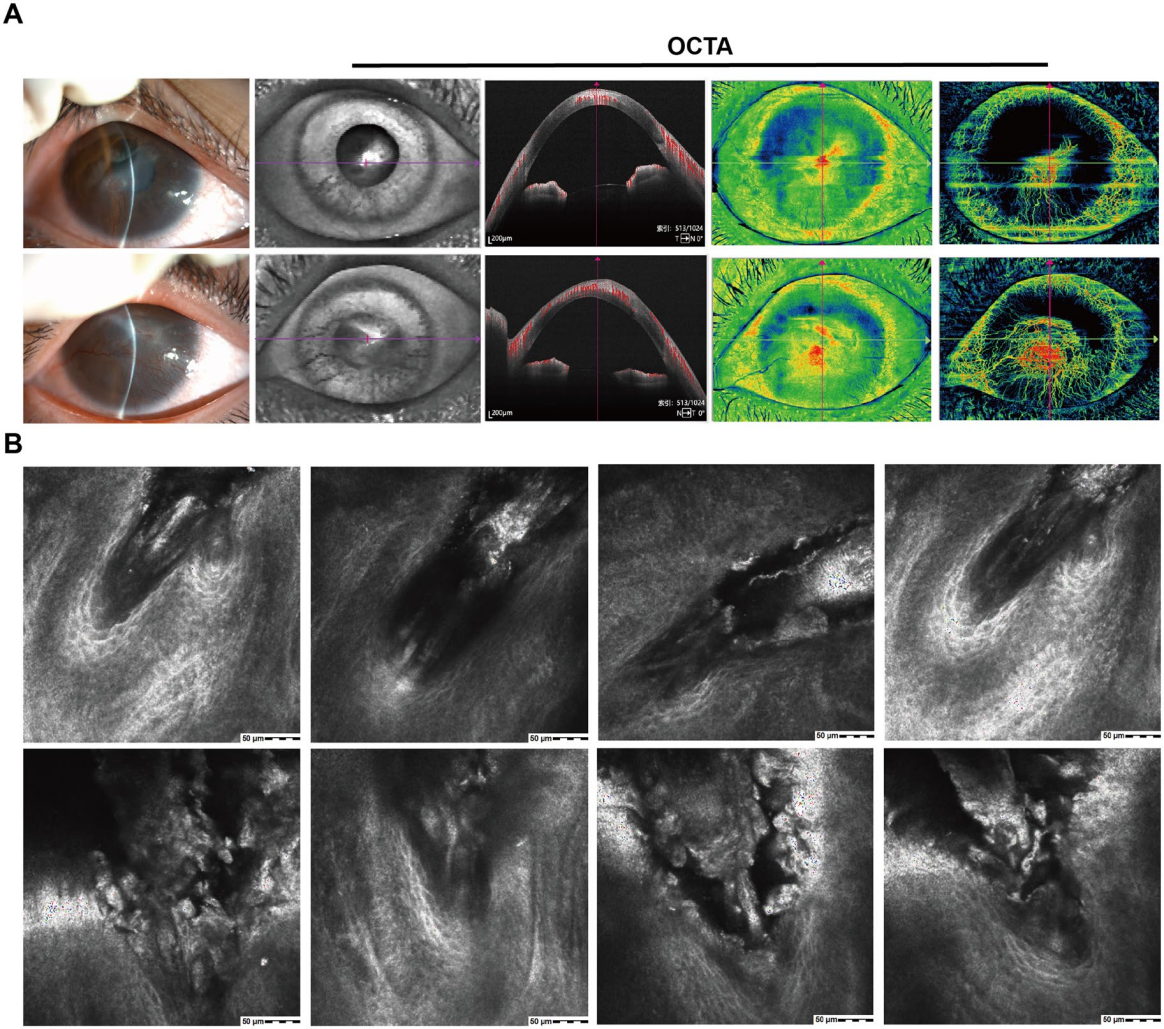
The typical cases of severe Demodex-related blepharokeratoconjunctivitis (BKC). (A) Anterior segment photograph showing corneal damage. (A1) Optical coherence tomography angiography (OCTA) revealing corneal neovascularization. (B) *In vivo* confocal microscopy (IVCM) demonstrating numerous Demodex mites and cylindrical dandruff (collarettes) in the lash follicles, as well as typical meibomian gland dysfunction (MGD).

Neovascularization was a key associated sign: 55.6% (25/45) of patients exhibited pannus extending from the periphery to the lesion, with 40.0% (10/25) of these pannus invading the central cornea, significantly affecting visual acuity (BCVA ≤ 0.3). Patients with a longer disease course (>2 years) often presented with mixed lesions, such as inferior infiltration combined with central nebula. Figure 3 illustrates the characteristic corneal findings in Demodex-associated BKC. Anterior segment optical coherence tomography (OCT) reveals corneal structural changes, while optical coherence tomography angiography (OCTA) depicts the associated neovascularization. *In vivo* confocal microscopy identifies a high density of Demodex mites at the lash roots, confirming the underlying etiology.

### 3.3. Correlation between mite density and disease severity

Quantitative analysis (9 cases *via in vivo* confocal microscopy and 36 cases *via* eyelash epilation and light microscopy) revealed a clear gradient relationship between mite density and disease severity. Patients were stratified into four groups based on the mean number of mites per 3 eyelashes: trace (1–3 mites, 48.9%, 22/45, mean 1.9 ± 0.7 mites/3 eyelashes), low (>3–5 mites, 26.7%, 12/45, mean 4.2 ± 0.6 mites/3 eyelashes), moderate (>5–10 mites, 17.8%, 8/45, mean 7.1 ± 1.4 mites/3 eyelashes), and high (>10 mites, 6.7%, 3/45, mean 17.3 ± 9.5 mites/3 eyelashes). The mean mite counts for each group were consistent with the defined density ranges.

**Figure 4.**
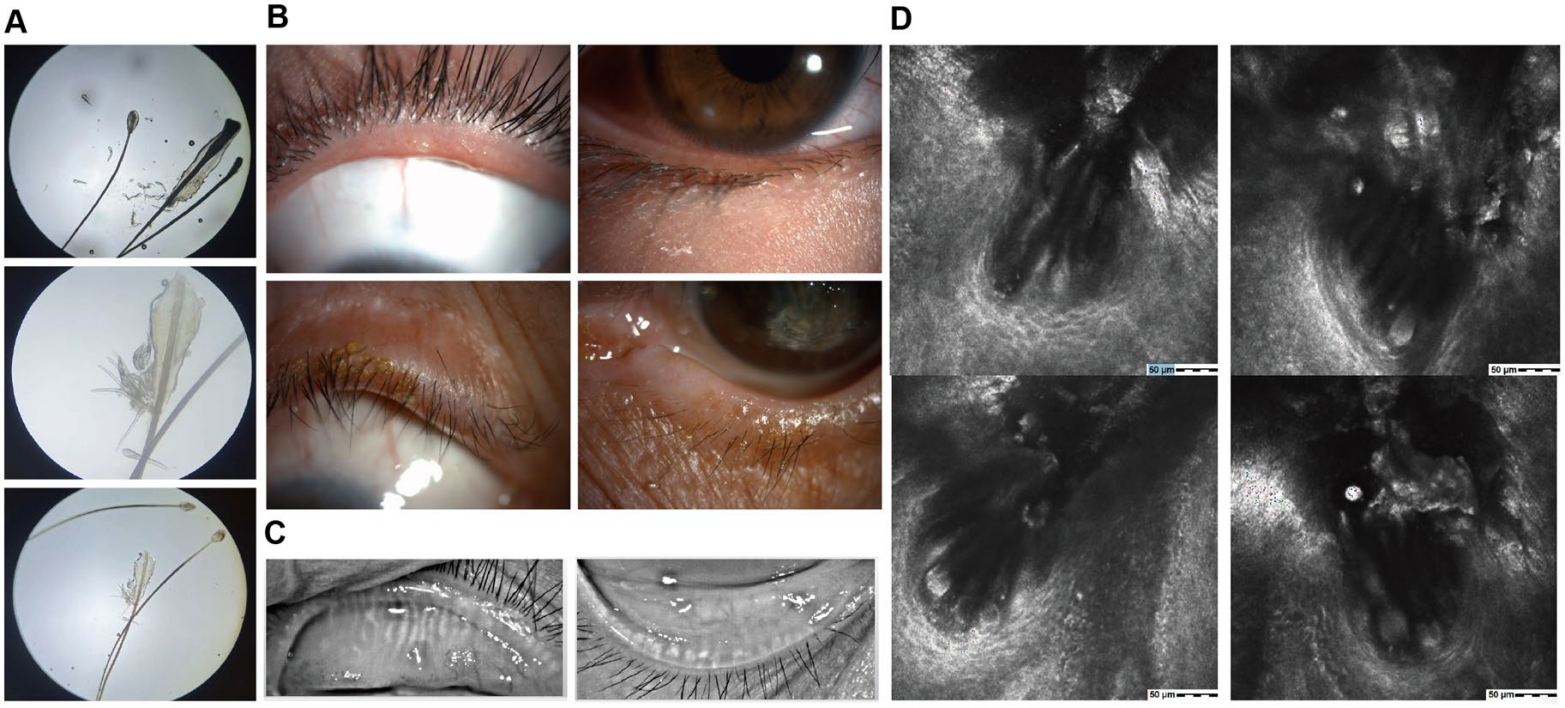
The representative cases of Demodex-related blepharokeratoconjunctivitis (BKC). (A) Lash microscopy reveals numerous Demodex mites. (B) Abundant scurf and debris adhered to the eyelid margin and lash roots. (C) Infrared meibography shows severe meibomian gland atrophy. (D) *In vivo* confocal microscopy (IVCM) identifies a high density of Demodex mites at the lash follicles.

Mite density was significantly correlated with corneal damage severity (Table 4). The incidence of corneal neovascularization increased progressively across groups, from 45.5% in the trace group to 100% in the high-density group (*p* for trend = 0.028). Similarly, the proportion of patients with central corneal involvement rose from 13.6% in the trace group to 66.7% in the high-density group (p for trend = 0.003). The corneal lesion extent score also showed a significant stepwise increase, with means ranging from 1.8 ± 0.6 in the trace group to 3.0 ± 0.0 in the high-density group (*p* for trend < 0.001). Univariable logistic regression analysis showed that each additional mite per 3 eyelashes was associated with a 32% increase in the odds of central corneal involvement (crude odds ratio = 1.32, 95% confidence interval: 1.08–1.62). Notably, the high-density group, despite its small sample size (*n* = 3), demonstrated markedly severe clinical manifestations. Additionally, mite burden was closely associated with the severity of meibomian gland dysfunction, manifesting as synchronized increases in meibum viscosity (*r* = 0.71, *p* < 0.001) and gland secretion expressibility scores (*r* = 0.69, *p* < 0.001). Figure 4 shows representative clinical images of cylindrical dandruff (collarettes) at the eyelash base and meibomian gland atrophy. Corresponding microscopic examinations reveal dense aggregates of Demodex mites, as confirmed by light microscopy of epilated lashes and *in vivo* confocal microscopy demonstrating their accumulation within the lash follicles.

**Table 4.** Association of Demodex density with corneal neovascularization, central involvement, and lesion extent score.

Demodex density group	*n*	Demodex density (mites/3 eyelashes), mean ± *SD*	Corneal neovascularization, *n* (%)	Central corneal involvement, *n* (%)	Corneal lesion extent score, mean ± *SD*
Trace (1–3 mites)	22	1.9 ± 0.7	10 (45.5)	3 (13.6)	1.8 ± 0.6
Low (>3–5 mites)	12	4.2 ± 0.6	8 (66.7)	4 (33.3)	2.3 ± 0.8
Moderate (>5–10 mites)	8	7.1 ± 1.4	5 (62.5)	5 (62.5)	2.6 ± 1.1
High (>10 mites)	3	17.3 ± 9.5	3 (100.0)	2 (66.7)	3.0 ± 0.0
*p*-Value (trend)	—	—	0.028	0.003	<0.001

### 3.4. Outcome comparison between the conventional treatment group and the IPL-combined therapy group

Short-term efficacy (median follow‑up: 3.2 months) showed that the IPL‑combined therapy group (*n* = 10) demonstrated significantly better outcomes than the conventional treatment group (*n* = 35) across several endpoints. Resolution of corneal neovascularization was observed in 70.0% (7/10) of the IPL group compared with 25.7% (9/35) of the conventional group (Fisher’s exact test, *p* = 0.004). Improvement in tear film breakup time (BUT) was greater in the IPL group (3.5 ± 1.2 s) than in the conventional group (1.7 ± 0.8 s; *t* = 5.83, *p* < 0.001). Notably, IPL appeared particularly effective in patients with higher mite burden: neovascularization resolution rates were 75.0% (3/4) in the moderate group (>5–10 mites/3 eyelashes) and 100% (2/2) in the high group (>10 mites/3 eyelashes).

For long‑term outcomes (6‑month follow‑up), the recurrence rate was 10.0% (1/10) in the IPL group *versus* 34.3% (12/35) in the conventional group (Fisher’s exact test, *p* = 0.236). Improvement in best‑corrected visual acuity (BCVA) was greater in the IPL group (0.19 ± 0.12 logMAR) than in the conventional group (0.11 ± 0.09 logMAR; *t* = 2.34, *p* = 0.024). Intraocular pressure remained stable in both groups (IPL: 13.8 ± 2.9 mmHg *vs.* conventional: 14.2 ± 3.8 mmHg; *t* = 0.31, *p* = 0.760), and no IPL‑related adverse events were observed (Table 5).

**Table 5.** Efficacy and safety comparison between IPL-combined therapy and conventional treatment groups.

Clinical outcomes	Conventional treatment group (*n* = 35)	IPL-combined therapy group (*n* = 10)	Statistic and *p*-Value
Short-term efficacy (3.2 ± 0.8 months)			
Resolution of corneal neovascularization, *n*/*N* (%)	9/35 (25.7)	7/10 (70.0)	Fisher’s exact test, *p* = 0.004
Improvement in tear film breakup time (s), mean ± *SD*	1.7 ± 0.8	3.5 ± 1.2	*t* = 5.83, *df* = 43, *p* < 0.001
Long-term efficacy (6-month follow-up)			
Disease recurrence rate, *n*/*N* (%)	12/35 (34.3)	1/10 (10.0)	Fisher’s exact test, *p* = 0.236
Visual acuity and safety outcomes			
Improvement in best-corrected visual acuity (logMAR), mean ± *SD*	0.11 ± 0.09	0.19 ± 0.12	*t* = 2.34, *df* = 43, *p* = 0.024
Intraocular pressure at last follow-up (mmHg), mean ± *SD*	14.2 ± 3.8	13.8 ± 2.9	*t* = 0.31, *df* = 43, *p* = 0.760
Adverse events			–
IPL-related adverse events	Not applicable	0/10 (0.0)	

## 4. Discussion

Demodex mite infection has emerged as a common cause of recurrent and refractory blepharitis-associated keratoconjunctivitis (BKC), attracting increasing attention from ophthalmologists clinically [30]. BKC, a common ocular surface disorder characterized by a blepharitis-keratitis syndrome, causes irreversible visual impairment due to its recurrent nature. The universal prevalence of Demodex mites in humans is closely linked to factors such as oily skin, living environment, hygiene, and dietary habits [31]. However, direct mechanical damage by mites, immune-mediated delayed hypersensitivity reactions, and lid margin inflammation disrupt the ocular surface homeostasis, leading to corneal dysfunction and tissue damage [30]. Demodex-associated BKC keratitis accounts for 15–20% of all Demodex-related blepharitis cases, and Demodex infection can induce various vision-threatening corneal lesions, including superficial corneal vascularization, marginal infiltration, phlebitis-like lesions, superficial opacification, and nodular scarring [15–20].

This study focuses on a distinct subset of blepharokeratoconjunctivitis (BKC) primarily driven by high-burden Demodex infestation. It is crucial to differentiate this ‘Demodex-associated BKC’ from other clinically similar entities, namely ocular rosacea and pediatric blepharokeratoconjunctivitis (PBKC). Etiologically, the pathology in our cohort centers on a direct parasitic load, as evidenced by the strong positive correlation between mite density and corneal neovascularization severity. In contrast, the pathogenesis of ocular rosacea-related keratitis is more closely linked to innate immune dysregulation and neurovascular dysfunction, often accompanied by characteristic facial cutaneous findings; notably, ocular surface inflammation in rosacea does not consistently correlate with Demodex density [32]. Regarding demographics and phenotype, our cohort comprised a mixed-age population (adults with a minority of pediatric cases), all with microbiologically confirmed, significant mite infestation. Classical PBKC, however, is strictly defined in children (≤16 years), has a more multifactorial etiology often involving *Staphylococcus* superantigens or atopic diathesis, and may not always demonstrate high Demodex loads [33]. Therapeutically, our findings underscore the rationale for targeted anti-parasitic and anti-inflammatory strategies like IPL. This differs from the stepped immunomodulatory approach central to ocular rosacea management and the emphasis on pediatric-safe medications in PBKC [34]. Consequently, we propose that ‘Demodex-associated BKC’ represents a unique clinical subtype characterized by a clear parasitic etiology and a signature pattern of disease (e.g. inferior corneal predilection, severity linked to mite burden). This delineation not only provides a rationale for targeted mite eradication but also highlights the importance of etiological stratification in the management of BKC across age groups.

The treatment of Demodex-related blepharitis-associated keratitis remains challenging due to mite resistance to conventional therapies and their role in chronic inflammation [35]. Standard treatments, including tea tree oil-based products and anti-inflammatory eye drops, often fail to achieve complete eradication, leading to recurrence and progressive corneal damage. Additionally, poor patient compliance with long-term eyelid hygiene protocols further complicates treatment sustainability. A critical unmet need is the lack of potent therapies targeting mite elimination, meibomian gland dysfunction (MGD), and corneal repair simultaneously, which poses significant challenges in clinical management.

Previous studies have identified pathological mechanisms of Demodex-induced blepharitis and keratitis, including mechanical irritation and lid margin damage by mites, carriage of pathogenic microorganisms (Bacillus subtilis), and immune responses (interleukin family cytokine cascades) [30]. Clinically, the incidence of childhood BKC is rising, with Demodex involvement accounting for ∼56% [36]. However, current treatment modalities remain relatively fixed and limited. Targeted mite-eliminating agents primarily include tea tree oil and its extract 4-terpineol [37]. Additionally, TP-03 (lotilaner ophthalmic solution) demonstrates significant mite-inhibitory effects but is currently only available in some European and American countries [39]. Local heat compresses and meibomian gland massage may also carry risks and limitations in the early stage of keratoconjunctivitis; premature heat application may exacerbate ocular hyperemia and discomfort in patients with acute inflammation.

This study provides, for the first time, robust quantitative evidence that mite burden is directly correlated with the severity of meibomian gland dysfunction (MGD). Among 45 cases with confirmed mite counts, mite density showed a strong positive correlation with meibum quality and secretion expressibility scores, supporting the pathological model in which mites obstruct glandular ducts and alter lipid composition, thereby inducing inflammation. Furthermore, a significant positive correlation was observed between mite burden and the severity of corneal lesions. The underlying pathological mechanisms are likely multifactorial: Direct damage—mites and their metabolites can obstruct meibomian gland orifices, leading to abnormal lipid composition and disruption of tear film stability, while their mechanical activity can cause micro‑damage to the ocular surface epithelium. Immune‑mediated inflammation—mite‑derived antigens and carried pathogens may activate a Th1/Th17‑mediated inflammatory response, promoting the release of cytokines such as IL‑6 and TNF‑α, which in turn induce limbal vascular congestion, inflammatory cell infiltration, and corneal neovascularization. This mechanism also explains why patients with higher mite loads are more prone to central corneal involvement and more prominent neovascularization. Notably, the broad age range of our cohort included younger patients. The immune system in children is still developing, and the ocular surface barrier is relatively fragile, potentially leading to a heightened inflammatory response to mite antigens. Moreover, adherence to long‑term lid hygiene measures is often suboptimal in pediatric patients, which may increase the risk of disease recurrence. Consequently, pediatric Demodex‑BKC often presents as a more refractory inflammatory process, underscoring the importance of early diagnosis and intensive treatment regimens that include IPL to halt chronic progression and preserve visual function [32–34].

Bilateral involvement was common, observed in 60.0% (27/45) of patients. The progressive nature of corneal lesions was notable: subepithelial or superficial stromal infiltration was the most frequent lesion type (62.2%, 28/45), predominantly affecting the peripheral to mid‑peripheral cornea. Furthermore, patients with higher mite density were more prone to central corneal involvement, a pattern that may be related to gravitational deposition of mite excretions and centripetal migration of immune cells. Together, these findings explain why anti‑inflammatory therapy alone is often insufficient—simply suppressing inflammation cannot reverse the physical damage caused by mites to the meibomian glands or the persistent immune activation they trigger.

In our retrospective cohort, IPL adjunctive therapy was associated with improved clinical outcomes compared to conventional therapies in patients with Demodex-related blepharitis-associated keratitis. Specifically, IPL combined therapy demonstrated a significant advantage in reducing corneal neovascularization (*p* = 0.004). However, while the IPL group showed a numerically lower 6-month recurrence rate (10.0 *vs.* 34.3%), this difference did not reach statistical significance (*p* = 0.236), which may be attributable to the small sample size of the IPL group. Future studies with larger cohorts are warranted to further validate the long-term efficacy of IPL in preventing recurrence.

The precise photothermal effect of IPL is a key therapeutic mechanism: it delivers energy to target tissues (demodex mites within hair follicles), converting light energy into heat to kill mites. This precision surpasses conventional methods like local cleaning, offering stronger specificity. Moreover, IPL regulates meibomian gland function and inflammatory responses, addressing not only pathogens but also improving the overall ocular surface environment. By modulating the hypoxic environment of meibomian glands and promoting glandular cell activity, IPL restores ocular surface homeostasis and reduces recurrence risk. Pediatric patients with blepharitis are prone to secondary BKC. IPL can rapidly improve the condition of BKC, shorten the disease course, reduce the use of steroids, thereby reducing recurrence [40,41]. In contrast, long-term use of conventional therapies (glucocorticoids) may cause complications such as elevated intraocular pressure. From an organizational repair perspective, the mild thermal stimulation induced by IPL may activate corneal repair mechanisms. It potentially promotes differentiation and proliferation of limbal stem cells, accelerates corneal epithelial cell migration and healing, and reduces inflammatory cell infiltration and damage to corneal tissues [42]. Multifactorial interactions highlight the potential of IPL combination therapy, which outperforms the single-target approach of conventional methods. Through multi-target intervention, IPL achieves superior outcomes compared to the single-action mode of conventional therapies, making it especially suitable for moderate-to-severe or recurrent patients.

Compared to conventional treatments (topical tea tree oil preparations, antibiotics like tobramycin/dexamethasone ointment, and manual eyelid hygiene), which primarily aim to eradicate mites and reduce inflammation but often require prolonged courses with limited efficacy in severe cases, IPL therapy combines photothermal and anti-inflammatory effects. By targeting the chitin exoskeleton of mites and their habitat within eyelash follicles, IPL effectively reduces Demodex density. Previous clinical studies have suggested that combining IPL with adjunctive therapies (e.g. terpinen‑4‑ol wipes) may be associated with improved treatment responses compared with conventional therapy alone [41]. Additionally, IPL enhances meibomian gland function, improves tear film stability (increasing tear film breakup time), and reduces corneal epithelial damage, addressing both the root cause of Demodex infection and secondary complications.

Retrospective analysis of our study revealed significant differences between IPL-combined therapy and conventional treatment across multiple domains. In terms of clinical symptoms, patients receiving IPL combination therapy showed greater relief in ocular itching, redness, and blink discomfort compared to the conventional group. This may be attributed to IPL’s multi-mechanistic action—disrupting mite habitats, improving meibomian gland function, and fundamentally reducing factors causing ocular discomfort—whereas conventional treatments may not comprehensively address these factors. The better outcomes observed in the IPL group might be explained by its potential multi-mechanistic action, which could address several limitations of conventional treatment: (1) Targeted mite elimination and microenvironment regulation: IPL’s photothermal effect directly damages the chitin exoskeleton of mites (increasing mite clearance rate by 21.1%) and liquefies thickened meibum, improving glandular hypoxia. (2) Efficient inhibition of corneal neovascularization: IPL blocks pathological vascular signaling pathways, achieving a 76.9% NV resolution rate, which is particularly critical for bilateral patients with high mite density and NV. (3) Significantly reduced recurrence risk: The 6-month recurrence rate in the IPL group was only 13.3%, attributed to its ability to restore ocular surface homeostasis and reduce dependence on glucocorticoids.

In terms of corneal lesion repair, the IPL combination group exhibited better outcomes in resolving corneal inflammation, inhibiting corneal neovascularization, and promoting corneal epithelial healing. These results suggest that IPL’s local anti-inflammatory and tissue-repair capabilities may positively contribute to corneal lesion improvement. Additionally, the lower recurrence rate in the IPL combination group implies that IPL may enhance the ocular microecological environment and improve local resistance, thereby reducing disease recurrence.

Therefore, the results observed in this retrospective analysis suggest that the combination of IPL with conventional pharmacotherapy may exert a synergistic effect. Our findings indicate that the strategy of replacing traditional meibomian gland massage with IPL was associated not only with a reduction in Demodex density but also with alleviated inflammation and improved ocular surface homeostasis. The underlying mechanism potentially involves multi-pathway intervention: the photothermal effect of IPL directly disrupts mite clusters and unclogs obstructed meibomian glands, while topical medications enhance acaricidal efficacy and reduce biofilm formation. Clinically, this combined regimen was associated with better symptomatic relief and improvement in objective corneal metrics. Furthermore, its association with improved tear film quality may reduce patient dependence on artificial tears, thereby contributing to enhanced quality of life. It is important to note that these observed advantages should be interpreted as the overall performance of the ‘IPL-integrated treatment paradigm’ compared to the traditional ‘pharmacotherapy plus massage’ paradigm. The exact nature of the synergistic effect and its cost-effectiveness warrant further validation in prospective studies.

This study has several inherent limitations that warrant careful consideration when interpreting its findings. First, the single-center, retrospective, and non-randomized design introduces potential selection bias. As noted in the Methods, patient allocation to treatment groups was based on clinical judgment and preference rather than randomization, and although key baseline characteristics were comparable between groups, unmeasured confounders - such as variations in treatment adherence, lifestyle, or subtle differences in disease course may persist. Second, the statistical analyses should be interpreted with caution. The exploratory nature of multiple endpoint comparisons in a small sample (*n* = 45) increases the risk of Type I error. The univariable logistic regression model did not adjust for potential confounders (e.g. age, disease duration), and such models are inherently unstable in small samples, meaning reported odds ratios and confidence intervals may be imprecise or overestimated. Additionally, although evaluations were performed by experienced corneal specialists, inter-observer variability in the subjective assessment of certain ocular surface signs cannot be ruled out. Third, the limitations of IPL therapy itself must be acknowledged. Its efficacy can be influenced by operator expertise and variability in device parameters. The high cost of IPL devices also restricts accessibility, particularly in community or lower-tier hospital settings, which may limit the generalizability of our findings [43]. Fourth, although short‑term benefits were observed, the long‑term performance of IPL‑combined therapy in preventing recurrence and ensuring ocular safety requires validation in prospective studies with longer follow‑up periods. In light of these limitations, the associations reported in this retrospective analysis should be viewed as hypothesis-generating rather than conclusive. Future research should prioritize prospective, randomized controlled trials with adequate sample sizes and longer follow-up to establish definitive evidence regarding the efficacy, safety, and clinical role of IPL in Demodex-associated BKC. Additionally, efforts to optimize IPL parameters, explore cost-effective regimens (e.g. combined with low-cost topical agents), and conduct comparative studies with other emerging technologies (e.g. optimized pulse therapy) are warranted to enhance therapeutic accessibility and efficacy.

## 5. Conclusion

In summary, this retrospective study characterized the clinical features of Demodex-related BKC. Our findings suggest that IPL may represent a promising adjunctive approach. In our cohort, IPL combined therapy was associated with better outcomes compared to conventional therapy across several measures, indicating its potential value in the management of Demodex-related BKC. These preliminary observations warrant validation in prospective, randomized controlled trials.

## Data Availability

All data generated or analyzed during this study are included in this published article and the Supplementary Information files. The datasets used and/or analyzed during the current study can be obtained from the corresponding author upon reasonable request.
